# Magnetically Recoverable δ‑FeOOH Particles for Multilayer Enzyme Immobilization and Surface-Induced Activity Tuning

**DOI:** 10.1021/acsomega.5c10160

**Published:** 2025-12-04

**Authors:** Francisco Lucas Chaves Almeida, Ederson Paulo Xavier Guilherme, Maria Isabel Rodriguez-Torres, Laura Rotilio, Magdalena Malankowska, Aliyeh Hasanzadeh, Bodil Fliis Holten, Suzana Siebenhaar, Lars Michael Skjolding, Jens P. Morth, John M. Woodley, Marcus Bruno Soares Forte, Elif Erdem

**Affiliations:** † Department of Chemical and Biochemical Engineering, 5205 Technical University of Denmark, 2800 Kgs Lyngby, Denmark; ‡ Bioprocess and Metabolic Engineering Laboratory, Department of Food Engineering and Technology, School of Food Engineering, 28132 University of Campinas, Rua Monteiro Lobato, 80—CEP, Campinas, 13083-862 São Paulo, Brazil; § Department of Biotechnology and Biomedicine, Technical University of Denmark, 2800 Kgs Lyngby, Denmark; ∥ Department of Chemistry Organic and Inorganic Chemistry, Technical University of Denmark, 2800 Kgs Lyngby, Denmark; ⊥ Department of Environmental and Resource Engineering, Technical University of Denmark, 2800 Kgs Lyngby, Denmark

## Abstract

Enzyme immobilization is an effective strategy to enhance enzyme stability, which remains a major drawback in biocatalysis. However, achieving high enzyme loading without loss of activity is still a key limitation of conventional support. Most supports that overcome these limitations, such as nano- and microparticles, involve high production costs. Therefore, a support that combines the advantages of high surface area, easy recovery, and low-cost production is still lacking in the literature. In this study, we present a low-cost, dual-function, magnetically recoverable support material, superparamagnetic δ-FeOOH (feroxyhyte) particles, that enhances enzyme activity and modulates protein structure both prior to, and following immobilization. We show that δ-FeOOH enables lipase immobilization at loadings exceeding 60 mg g^–1^ while maintaining activity and structural integrity. Circular dichroism and fluorescence analyses reveal support-induced conformational changes, decreased α-helicity and increased β-sheet content, that do not impair enzymatic performance. Zeta potential analysis further confirms progressive surface saturation and multilayer formation, with continued adsorption beyond ∼40 mg g^–1^, without functional decline. Notably, both lipase and NADH oxidase (L*p*NOX) exhibit up to 1.3-fold activity enhancement in the presence of δ-FeOOH, even in the absence of covalent binding, suggesting a surface-induced activation mechanism. Together, these findings establish δ-FeOOH as a high-capacity, structurally tunable enzyme support. Its ability to promote both immobilization and functional enhancement makes it a promising platform for next-generation biocatalysts in continuous, high-density, and multienzyme systems.

## Graphical abstract

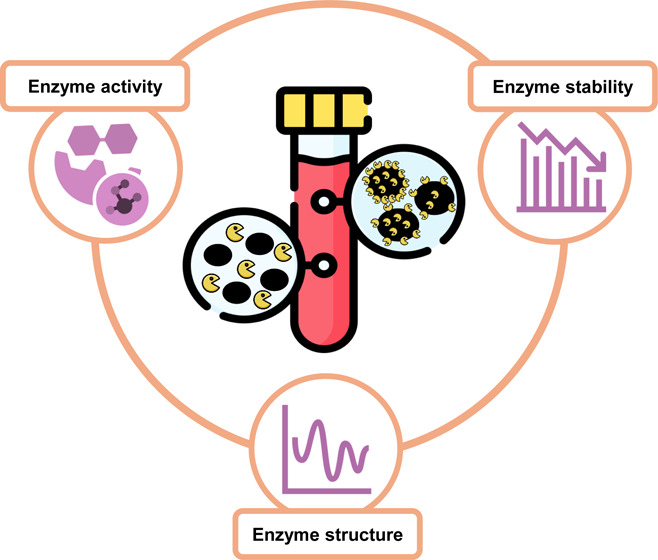

## Scientific Highlights

- Superparamagnetic δ-FeOOH particles function as dual-role support, facilitating enzyme immobilization and surface-induced activation.
- Lipases were immobilized at high loading densities (>60 mg g^–1^ support) with satisfatory catalytic efficiency or conformational stability.
- Zeta potential analysis and spectroscopic data confirmed multilayer adsorption beyond monolayer saturation (∼40 mg g^–1^).
- Interaction with δ-FeOOH modulated enzyme secondary structure, decreasing α-helical and increasing β-sheet content.
- Incubation with δ-FeOOH enhanced the specific activity of both lipase and L*p*NOX by up to 1.3-fold, independent of covalent binding.

## Introduction

The industrial implementation of biocatalysis relies heavily on enzyme immobilization, which enhances enzyme stability, enables reuse, and simplifies separation from reaction mixtures. Enzymes such as lipases, oxidases, and flavin-dependent dehydrogenases are widely applied due to their high catalytic activity and selectivity under mild conditions.^1^ However, their practical use is often limited by poor operational stability, susceptibility to inactivation, and challenges in recovery. Developing platforms that combine high enzyme loading with sustained activity remains a key challenge in the design of efficient immobilized biocatalysts.^2–4^

Superparamagnetic δ-FeOOH (feroxyhyte) particles present a promising, yet underexplored, support material for enzyme immobilization. These particles offer a high surface area for dense enzyme adsorption and possess magnetic properties that enable rapid, noninvasive recovery using external magnetic fields. Such features are advantageous for continuous-flow processes and recyclable catalytic systems.^5–9^ Despite these benefits, the impact of δ-FeOOH on enzyme structure and function has not been systematically evaluated.

Lipases (triacylglycerol acyl hydrolases, EC 3.1.1.3) are among the most commercially relevant enzymes, with applications across food processing (e.g., flavor esters, aromatic esters), pharmaceuticals (e.g., emulsifiers, cosmetics oils), and biofuels (e.g., biodiesel).^10^ Their global market share continues to grow, driven by their versatility in catalyzing esterification, transesterification, and hydrolytic reactions.^10^ Structurally, many lipases exhibit interfacial activation, a mechanism regulated by a mobile lid domain that shields the active site, in the absence of an interface. Upon encountering a hydrophobic interface, the lid undergoes a conformational change, exposing the active site and significantly enhancing catalytic activity.^11–14^ This behavior is essential for lipase function but also makes enzyme-support interactions a critical factor during immobilization, as support properties can influence lid dynamics and enzyme accessibility.

In addition, oxygenases, more specifically NADH oxidases (EC 1.6.3.4) such as LpNOX, are also widely used enzymes in biocatalytic oxidation processes, mainly due to their ability to regenerate the expensive cofactor NAD­(P)^+^. Biocatalytic oxidations employing these enzymes have been applied in the production of important pharmaceutical compounds such as belzutifan and esomeprazole, in the oxidation of hydroxylactols to the corresponding hydroxylactones, and in the synthesis of fragrances like undecavertol. However, like lipases and most other enzymes, they also exhibit low stability.^15,16^

Different types of particle-based support have been shown to affect enzyme orientation, packing density, and structural conformation, all of which impact catalytic performance. Liu et al.^17^ reported that silver nanoparticles induced conformational changes in catalase, leading to reduced activity, while superoxide dismutase remained unaffected. Saha et al.^18^ demonstrated that trypsin activity was modulated by enzyme density and monolayer formation on copper sulfide nanoparticles. More recently, Luo et al.^19^ highlighted that the orientation and surface conformation of immobilized enzymes can exert a greater influence on catalytic efficiency than structural changes alone, although this remains poorly understood.

Despite these reports, it is clear that much remains to be explored in this field, as each enzyme-particle system exhibits unique characteristics, and even subtle differences in interaction (or lack thereof) can lead to distinct behaviors. Furthermore, no studies have thoroughly investigated the effect of magnetic particles, particularly low-cost materials such as δ-FeOOH, on fundamental and industrially relevant enzymes such as lipases and LpNOX. To address these limitations and gain a deeper understanding of enzyme-support interactions, we investigated δ-FeOOH magnetic particles as a novel support for immobilization. This study contributes to the literature not only by providing insights into how enzyme-particle interactions influence catalytic activity and immobilization efficiency but also by offering new perspectives on enzyme structural behavior.

To better achieve it, a δ-FeOOH was synthesized, and two model lipases and an oxidase were selected for this study: LipA, which lacks a lid domain, and Lip3, which possesses a canonical lid, L*p*NOX, a water-forming NADH oxidase. This design enables a direct assessment of how the presence of a lipase lid influences immobilization-induced structural changes and enzymatic performance while showing the applicability of the support with another enzyme. Our results show that δ-FeOOH supports allow for high enzyme loading (>60 mg g^–1^) while maintaining activity. Spectroscopic analysis revealed a shift from α-helical to β-sheet content in both enzyme types upon immobilization, yet catalytic activity was preserved. Notably, both lipase and L*p*NOX exhibited enhanced activity, indicating that δ-FeOOH supports are compatible with multienzyme redox systems. These findings position δ-FeOOH as a scalable, magnetically recoverable, and biocompatible support material, with significant potential for use in continuous biocatalysis and cofactor regeneration processes.

## Experimental Section

### Chemicals

Ethanol absolute (CAS no. 64-17-5, VWR chemicals), 4-nitrophenil butyrate (CAS no. 2635-84-9, Merck Life Science A/S), 4-nitrophenol (CAS no. 100-02-7, Sigma-Aldrich), Iron­(III) sulfate heptahydrate (CAS no. 7782-63-0, Sigma-Aldrich), Tetraethyl orthosilicate—TEOS (CAS no. 78-10-4, Sigma-Aldrich), Glutaraldehyde solution 25% (CAS no. 111-30-8, Sigma-Aldrich), (3-Aminopropyl) triethoxysilane—APTES (CAS no. 919-30-2, Sigma-Aldrich), Hydrogen peroxide solution 30% (CAS no. 7722-84-1, Sigma-Aldrich) were used in this study.

### Methods

#### Lipase Production and Purification

##### Plasmid Transformation and Overnight Cultures

The plasmids and strains used in this study are listed in Table 1. Transformation was performed following the GenScript Biotech protocol. *Escherichia coli* strains (BL21 (DE3) Star and SHuffle) were thawed on ice for 10 min, and 2 μL of plasmid DNA (1 pg–100 ng) was gently mixed with competent cells. The mixture was incubated on ice for 30 min, heat-shocked at 42 °C for 30 s, and returned to ice for 5 min. Subsequently, 950 μL of LB medium was added, and cells were incubated at 30 °C with shaking (250 rpm, 1 h). After recovery, 100 μL of the transformation mix was plated on LB agar and incubated at 25 °C for 48 h. Colonies were picked and inoculated into 5 mL LB in 15 mL tubes and cultured overnight at 37 °C, 150 rpm.

**Table 1:** Description of Supports, Lipases and Albumin in Free and Immobilized forms and Codes Used in the Text

description	code
Albumin	Alb
Lipase A	LipA
Lipase 3	Lip3
δ -FeOOH@SiO2	S1
δ -FeOOH@SiO_2_@NH_2_-GL0.5	S2
δ -FeOOH@SiO_2_@NH_2_-GL1	S3
δ -FeOOH@SiO_2__Alb	S1_Alb
δ -FeOOH@SiO_2_@NH_2_-GL0.5_Alb	S2_Alb
δ -FeOOH@SiO_2_@NH_2_-GL1_Alb	S3_Alb
δ -FeOOH@SiO_2__LipA	S1_LipA
δ -FeOOH@SiO_2_@NH_2_-GL0.5_LipA	S2_LipA
δ -FeOOH@SiO_2_@NH_2_-GL1_LipA	S3_LipA
δ -FeOOH@SiO_2__Lip3	S1_Lip3
δ -FeOOH@SiO_2_@NH_2_-GL0.5_Lip3	S2_Lip3
δ -FeOOH@SiO_2_@NH_2_-GL1_Lip3	S3_Lip3

##### Whole-Cell Cultivation

For production, 2 mL of overnight preculture was used to inoculate 500 mL of Terrific Broth (TB) in 2 L baffled flasks. Cultures were grown at 37 °C, 150 rpm, until OD_600_ reached 0.5–0.9 (e.g., BL21 Star_Lip3:0.93; SHuffle_Lip3:0.61). Protein expression was induced with 0.5 mM IPTG and cultures were incubated at 15 °C for 19 h. Final OD_600_ values ranged from 14 to 18, depending on strain and construct.

##### Cell Lysis, Purification, and SDS-PAGE

Cells were lysed using a high-pressure homogenizer (GEA Panda Plus 2000) at 900–1000 bar (2 passes per sample). His-tagged proteins were purified under native conditions using Ni-NTA resin (HisPur, Macherey-Nagel) following the manufacturer’s protocol. All buffers were prepared and stored at room temperature (∼17 °C); purification steps were performed at room temperature with enzymes kept on ice throughout. Imidazole was removed from purified fractions using Zeba spin desalting columns (Thermo Scientific) equilibrated with phosphate buffer (100 mM, pH 7.0). Protein concentration was determined by UV absorbance, and purity was assessed by SDS-PAGE (10%, 110 V), followed by Coomassie staining and imaging with a Bio-Rad system.

#### Synthesis and Functionalization of δ-FeOOH Magnetic Particles

##### Synthesis of δ-FeOOH Particles

Magnetic δ-FeOOH was synthesized based on the method of Guilherme et al.^9^ Briefly, 10 g of FeSO_4_·H_2_O was dissolved in 250 mL of distilled water under magnetic stirring. Precipitation was induced by the dropwise addition of 100 mL NaOH (2 M) under stirring for 30 min, followed by oxidation with 5 mL H_2_O_2_ and continued stirring for 10 min. The resulting particles were washed three times with deionized water before functionalization.

##### Support Functionalization

To obtain δ-FeOOH@SiO_2_@NH_2_, 5 g of washed δ-FeOOH was suspended in 5 mL distilled water and 25 mL ethanol, and stirred for 30 min. Then, 10 mL NH_4_OH and 5 mL TEOS were added and stirred for 4 h. This was followed by the addition of 5 mL APTES and continued stirring for 16 h. After that, functionalized particles were washed three times with water. Then, part of the particles was separated and stored in phosphate buffer (100 mM, pH 7.0), while the remainder was treated with glutaraldehyde. To generate covalently active supports treated with glutaraldehyde, δ-FeOOH@SiO_2_@NH_2_ (S1) particles were treated with either 0.5% or 1.0% glutaraldehyde solutions (v/v) for 4 h under agitation. The activated supports (δ-FeOOH@SiO_2_@NH_2_–S3 and S4) were washed three times with deionized water and stored in phosphate buffer (100 mM, pH 7.0) until use.

#### Preparation of Physical Mixtures

Lipase, LpNOX and bovine serum albumin (BSA) stock solutions were prepared in phosphate buffer (100 mM, pH 7.0) at a final protein concentration of 0.3 mg mL^–1^. Supports were added to the protein solution at final concentrations ranging from 0 to 1870 μg mL^–1^. Mixtures were incubated on ice without agitation for 1 h prior to structural or activity analysis.

#### Protein Immobilization

Protein immobilization was carried out using three different δ-FeOOH-based magnetic supports via both adsorption and covalent coupling methods (Table 1). For each immobilization experiment, proteins were added to a phosphate buffer (100 mM, pH 7.0) containing the support at an initial ratio of approximately 150 mg protein per gram of support. The immobilization procedure was conducted in 50 mL centrifuge tubes. Protein-support mixtures were incubated at 30 °C in a shaker under continuous agitation (250 rpm) for 2 h. Following incubation, supports were separated magnetically and washed three times with fresh buffer to remove unbound protein. In the washing step, fresh buffer was added, and the tubes were gently mixed and centrifuged. The supernatant was then collected. Protein concentrations in the supernatants and wash solutions were quantified using the Bradford assay and UV absorbance (Nanodrop, 280 nm) as described before. The amount of protein immobilized on the support was calculated based on protein depletion from the solution, using the following equation.^20^

$$Immobilization\;efficiency\;\left(\%\right)=\left(\frac{m-C\times V}{m}\right)\times 100\%$$

where *m* (mg) is the initial mass of protein added, *C* (mg mL^–1^) is the protein concentration in the supernatant and washes, and *V* (mL) is the total volume of supernatant and wash buffer collected.

#### Particle Characterization

##### Surface Morphology and Composition

The morphology of unloaded and protein-loaded δ-FeOOH particles was analyzed by scanning electron microscopy (SEM) using a Prisma E-SEM (Thermo Fisher Scientific) operated at 5–10 kV. Samples were first centrifuged to remove excess liquid, then mounted on carbon tape and left to dry at room temperature. Surface functionalization at each step of particle synthesis was monitored by Fourier-transform infrared (FTIR) spectroscopy using the Attenuated Total Reflectance (ATR) mode, in the range of 4000–600 cm^–1^, with an Invenio-S spectrometer (Bruker). Specific surface area was determined from nitrogen adsorption–desorption isotherms using a Micromeritics ASAP 2020 system at 77 K and estimated based on Brunauer–Emmett–Teller (BET) theory. For FTIR and s-Bet analyses, samples were dried in an oven overnight at 60 °C. Zeta potential and hydrodynamic diameter were measured using dynamic light scattering (DLS) on a Zetasizer Nano ZS (Malvern Instruments, UK) in a cuvette containing phosphate buffer (100 mM, pH 7.0) at room temperature.

#### Protein Structural Analysis

##### Endogenous Fluorescence Spectroscopy

Fluorescence emission spectra were recorded using a Jasco FP-8200 spectrofluorometer. Protein samples (0.01 mg mL^–1^ in 100 mM phosphate buffer, pH 7.0) were excited at 280 nm, and emission was recorded from 275 to 450 at 1 nm intervals. Results represent the average of at least two independent spectra. Phosphate buffer and buffer + support were used as blanks in all measurements. All samples were kept on ice throughout the entire analysis and were gently mixed by inverting the Eppendorf tubes.

##### Circular Dichroism (CD) Spectroscopy

CD spectra were acquired using a Jasco J-1500 spectropolarimeter equipped with a Peltier-controlled cuvette holder. Protein solutions (0.3 mg mL^–1^ in 100 mM phosphate buffer, pH 7.0) were analyzed in a 0.1 cm path length cuvette at 20 °C. Spectra were recorded from 195 to 250 nm at a scan rate of 100 nm min^–1^ with a bandwidth of 1 nm. Each spectrum represents the average of three consecutive scans. Buffer and buffer + support controls were subtracted from all spectra. All samples were kept on ice throughout the entire analysis and were gently mixed by inverting the Eppendorf tubes.

##### Thermal Unfolding (nDSF)

Thermal unfolding of proteins was measured using a Prometheus Panta instrument (NanoTemper, Germany), combining nanodifferential scanning fluorimetry (nDSF), turbidity, and DLS. Samples were loaded into the capillary system and heated from 20 to 90 °C at a rate of 1 °C min^–1^, then cooled back to 20 °C at the same rate. The unfolding transition was monitored by tracking the fluorescence emission ratio at 350/330 nm (F_350_/F_330_) upon excitation at 280 nm.

#### Protein Quantification

Protein concentrations were determined using both UV absorbance (Nanodrop OneC, Thermo Scientific) and Bradford protein assay. Extinction coefficients and molecular weights (Table S1) specific to each protein were used to calculate concentrations at 280 nm. Bradford assays were performed in triplicate on a microplate reader (SpectroStar Nano, BMG Labtech) using bovine serum albumin as standard.

#### Lipase Activity Assay

Lipase activity was determined by using *p*-nitrophenyl butyrate (pNPB) or *p*-nitrophenyl palmitate (pNPP) as substrates. The reaction mixture (975 μL total) contained 100 μL lipase solution (0.3 mg mL^–1^ LipA; 0.62 mg mL^–1^ Lip2 BL21-Star; 0.43 mg mL^–1^ Lip2 SHuffle; 0.1 mg mL^–1^ Lip3), 25 μL substrate (20 mM in ethanol), and 850 μL phosphate buffer (100 mM, pH 7.0). The mixture was incubated at 25 °C for 2 min under agitation (1000 rpm), and the reaction was stopped by adding 1 mL absolute ethanol. Absorbance of released *p*-nitrophenol was measured at 410 nm using a Shimadzu UV-1800 spectrophotometer. Activity was calculated using a standard curve. One unit (U) of lipase activity was defined as the amount of enzyme that produces 1 μmol of *p*-nitrophenol per minute at 25 °C, pH 7.0. For thermal stability tests, enzymes were preincubated at 50 °C for 2 h and then assayed under the same conditions.

#### LpNOX Activity Assay

NAD­(P)H oxidase (LpNOX) activity was measured according to the method of Wang and Woodley.^21^ The reaction mixture (1.0 mL) contained 42 μL LpNOX (0.3 g L^–1^), 150 μL NADPH (1 g L^–1^), and 808 μL phosphate buffer (50 mM, pH 7.0). The decrease in NADPH absorbance at 340 nm was monitored over 120 s at 25 °C using a Shimadzu UV-1800 spectrophotometer in a 1 cm path length semimicro quartz cuvette. The extinction coefficient ε_340_ = 6.22 mM^–1^ cm^–1^ was used to calculate activity. One unit (U) of NOX activity was defined as the amount of enzyme that oxidizes 1 μmol of NADPH per minute at 25 °C, pH 7.0.

## Results and Discussion

### Expression and Production of Enzymes

To investigate the structural and functional effects of δ-FeOOH magnetic particles on lipase immobilization, we selected two model enzymes among three lipases reported in the literature (Table S1) with distinct architectures: LipA, a lipase variant lacking a lid domain, and Lip3, which possesses a canonical lid associated with interfacial activation. The lid domain modulates access to the active site and is known to undergo conformational transitions in response to hydrophobic interfaces. This system was designed to assess whether lid-associated dynamics influence structural rearrangement and catalytic performance upon immobilization. Recombinant expression of both lipases and L*p*NOX was performed in *E. coli* BL21 Star and Shuffle strains (Table S2). Shuffle strain possesses an oxidative cytoplasm that facilitates disulfide bond formation, while BL21 Star is optimized for high-yield expression of soluble proteins. Both strains produced soluble, well-folded enzymes, as confirmed by SDS-PAGE and soluble fraction analysis (Figure S1a–c). Due to higher expression levels, BL21 Star was selected for preparative-scale production and used for all subsequent immobilization studies. To assess the role of protein surface properties on δ-FeOOH binding, bovine serum albumin (BSA) was included as a nonenzymatic, hydrophilic control. BSA is a well-characterized, globular protein lacking catalytic activity toward lipid substrates. Its inclusion enabled differentiation between lipase-specific effects and general protein adsorption. All proteins were used in their native recombinant form to preserve structural and functional integrity during immobilization.

### Production and Characterization of Magnetic δ-FeOOH Particles

To support the immobilization of the produced lipases, we next characterized the structural and chemical properties of the δ-FeOOH-based magnetic particles used as immobilization supports (Figure 1a). All supports show size around 2500 nm (Table S3). The morphology and physicochemical properties of the synthesized magnetic particles were characterized to assess their suitability for enzyme immobilization. Scanning electron microscopy (SEM) revealed that δ-FeOOH particles form compact, irregular aggregates with a strong tendency to agglomerate, likely due to interparticle magnetic interactions (Figure 1c). Similar morphological features have been reported for δ-FeOOH synthesized for peroxidase immobilization.^8^ Moreover, to show that the particles can be used for immobilization, we fist tested them with BSA and SEM images revealed the coverage of particles with BSA (Figure 1c—BSA images). FTIR spectroscopy (600–4000 cm^–1^) was used to identify functional groups present on both native (ADS) and chemically modified particles (S1–S3) (Figure 1b). The presence of characteristic bending vibrations in the 849–1271 cm^–1^ range (regions 4 and 5) for ADS confirms the successful synthesis of δ-FeOOH, consistent with prior studies.^22^ Functionalization with tetraethyl orthosilicate (TEOS), (3-aminopropyl)­triethoxysilane (APTES), and glutaraldehyde was verified in S1 to S3 particles, when compared with ADS, by the appearance of diagnostic bands corresponding to Si–O–Si, Si–O–Fe, and Si–O–H linkages, as well as sulfur–oxygen stretching (regions 2, 4, and 5).^23,24^

**Figure 1:**
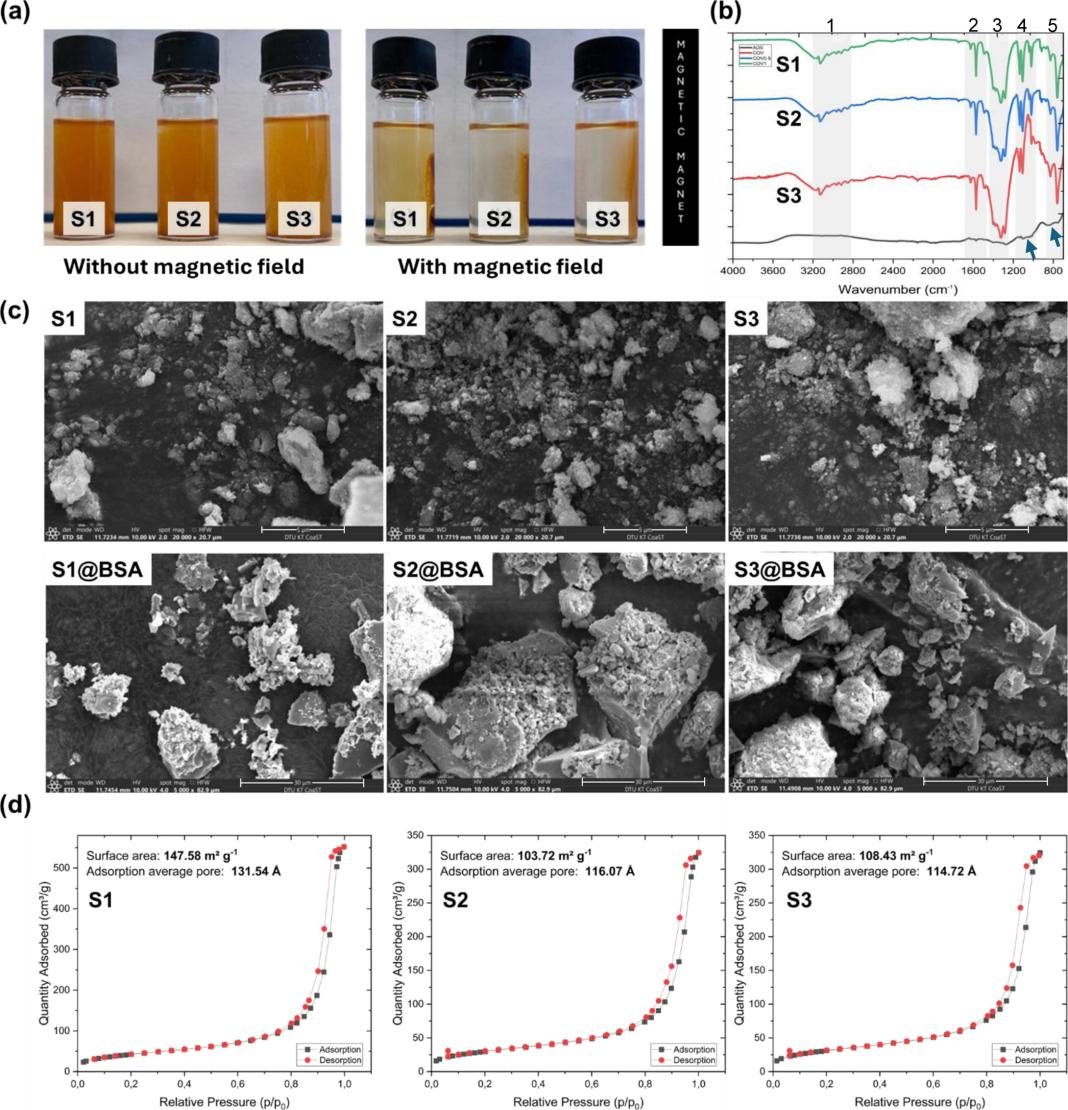
Characterization of δ-FeOOH-based magnetic supports. (a) Supports in solution S1 represents δ -FeOOH@SiO_2_, S2 δ -FeOOH@SiO_2_@NH_2_-GL0.5, and S3 δ -FeOOH@SiO_2_@NH_2_-GL1 respectively. (b) Fourier-transform infrared (FTIR) spectra of supports highlighting functional group modifications due to silanization and glutaraldehyde coupling. (c) Scanning electron microscopy (SEM) images showing particle morphology and surface texture. S1–S3 (20,000× magnification; scale bar = 5 μm); S1@BSA–S3@BSA (5000× magnification; scale bar = 30 μm) (d) Nitrogen adsorption/desorption isotherms used for Brunauer–Emmett–Teller (BET) surface area and porosity analysis of supports showing mesoporous profiles consistent with IUPAC type V isotherms. Source: Photos taken by the authors.

Surface properties were also analyzed using nitrogen adsorption–desorption isotherms and the Brunauer–Emmett–Teller (BET) method (Figure 1d). The specific surface areas of the particles ranged from 103.72 to 147.58 m^2^ g^–1^, aligning well with reported values for β-FeOOH and δ-FeOOH materials, which span 16–330 m^2^ g^–1^. The isotherms were classified as type V, a profile characteristic of mesoporous materials with moderate adsorbate–adsorbent interactions. Consistent with IUPAC definitions, all samples exhibited average pore diameters within the mesoporous range of 2–50 nm.^25^ It is interesting to point out that a range from 16 m^2^ g^–1^ to 330 m^2^ g^–1^ can be found in the literature for β-FeOOH structures, which are similar to the ones in this study.^26^ We observed an increase from around 60 m^2^ g^–1^ (ADS surface area) to 100 m^2^ g^–1^ in surface area after coating using TEOS (SiC_8_H_20_O_4_) and APTES (C_9_H_23_NO_3_Si), which may be associated with the fact that it can lead to a formation of silica shell around the particles. This silica shell can contribute to the overall surface area, mainly due to the presence of pores. This phenomenon aligns with findings by Reczyńska et al.,^27^ who reported a remarkable increase in surface area, from 7.54 ± 0.02 m^2^ g^–1^ to 101.3 ± 2.8 m^2^ g^–1^, following the coating of superparamagnetic iron (II, III) oxide nanoparticles with a layer of mesoporous silica coating. These results confirm that the synthesized particles exhibit the necessary surface area, porosity, and chemical functionality to serve as effective supports for enzyme immobilization.

### Effect of Magnetic Particles on Enzyme Performance

Before evaluating enzyme immobilization, we first investigated whether δ-FeOOH magnetic particles alone, without covalent attachment, or adsorption, could influence enzyme structure and activity. Previous studies have shown that enzymes typically adopt a low energy conformation under stable conditions. However, external stimuli such as magnetic fields or particle surfaces can shift this equilibrium, promoting more flexible or catalytically active states.^28^ In addition, particles introduced into biological environments can interact with proteins and other biomolecules to form a dynamic “biological identity,” which may alter both particle surface properties and the behavior of surrounding macromolecules.^29^ These effects remain largely unexplored in the context of enzyme activation in biocatalysis. To assess these potential influences, δ-FeOOH particles were incubated at varying concentrations (up to 1860 μg mL^–1^) with 0.3 mg of lipase in solution. Protein conformational changes were monitored by circular dichroism (CD), which reveals secondary structure content, and tryptophan fluorescence spectroscopy, which provides information on tertiary structure and the local environment of aromatic residues. At concentrations ≥370 μg mL^–1^, a notable decrease in fluorescence intensity was observed for both LipA and Lip3 (Figure 2a–c), indicating perturbation of the tertiary structure. Iosin et al.^30^ reported a decrease in fluorescence with an increase in gold nanostructure concentration, as well as Dyawanapelly et al.^31^ and Dyawanapelly et al.^32^ studying lipase and functionalized iron nanoparticles. It is also important to note that Iosin et al.^30^ demonstrated that the complete binding of nanoparticles to enzymes leads to nearly complete quenching of fluorescence.

**Figure 2:**
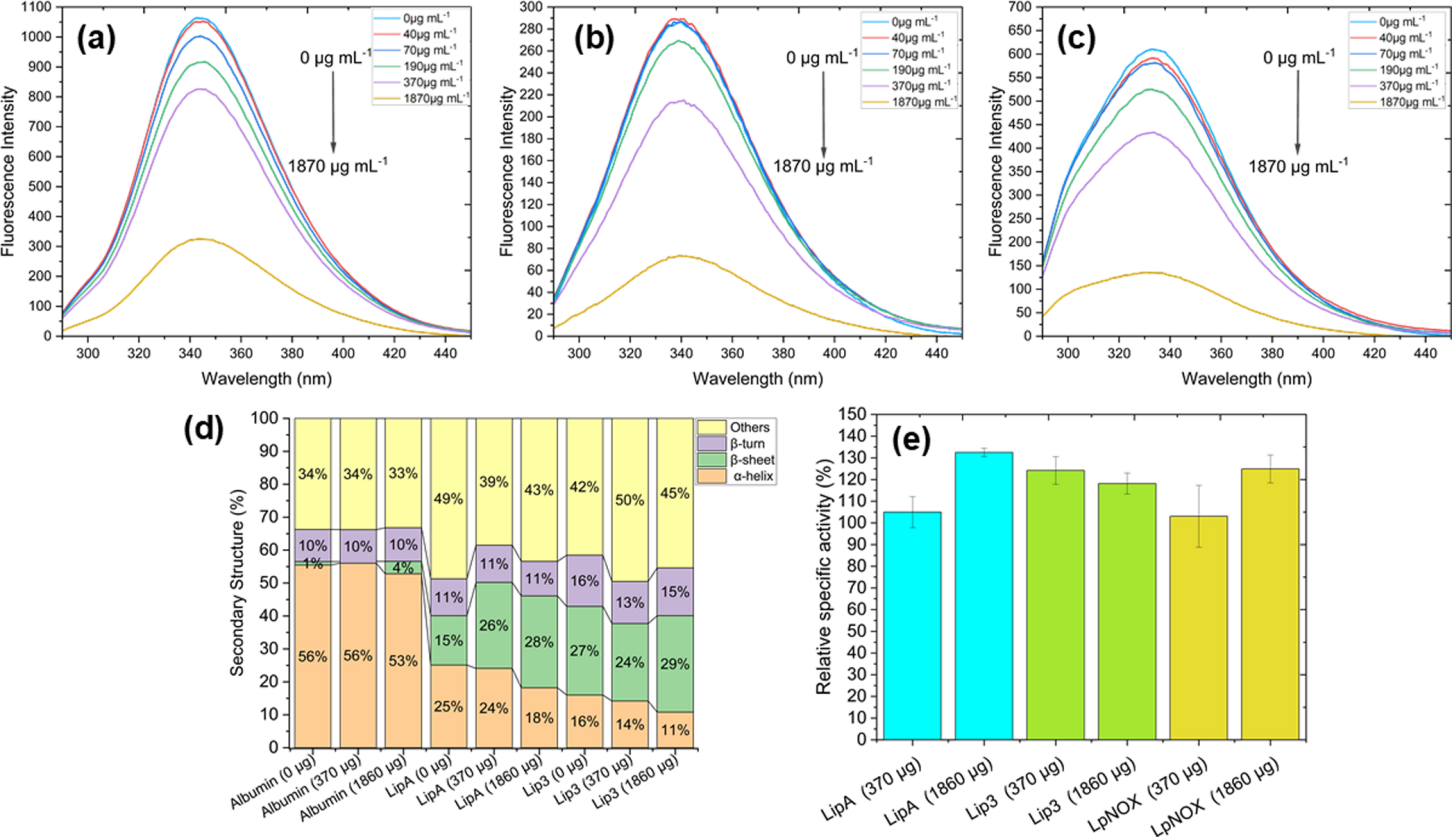
Effect of magnetic supports on enzyme structure and activity. (a–c) Intrinsic tryptophan fluorescence spectra of bovine serum albumin (a), LipA (b), and Lip3 (c) following incubation with increasing concentrations of δ-FeOOH particles (0–1860 μg mL^–1^), showing tertiary structure perturbations. (d) Circular dichroism analysis of secondary structure changes in Lip3 and LipA in the presence of magnetic support (0, 370, and 1860 μg mL^–1^). (e) Relative specific activity of lipases at different support concentrations. Data indicate support-induced structural rearrangements can correlate with enhanced catalytic performance at moderate support concentrations. *Relative specific activity calculated as enzyme specific activity (370 or 1860 μg)/ enzyme specific activity (0 μg) × 100.

CD spectroscopy is a powerful technique for studying the interaction of the protein with other molecules in solution or absorbed on other molecules^33^ since we applied it to understand the effect of mixing δ-FeOOH structures and lipases/albumin. CD analysis revealed a concurrent reduction in α-helical content and an increase in β-sheet structures (Figure 2d), consistent with partial conformational rearrangement. de Barros et al.^34^ reported that CALB remained almost unchanged after being absorbed into AuNPs (citrate-stabilized gold nanoparticles), since the signal at 222 nm, characteristic of α-helical proteins remained unchanged, indicating that an order–disorder (unfolding) process has not occurred. However, Suo et al.^35^ observed results like our study, and they reported that the decrease in α-helix content may lead to the opening of the helical lid of lipase, while the increase in β-sheet results in a more rigid structure of the immobilized lipase, which may lead to enhanced thermal stability and tolerance to denaturants.

These structural changes correlate with a significant enhancement in enzyme activity. At the highest particle concentrations tested, LipA exhibited a 1.3-fold increase in specific activity, while Lip3 showed a 1.2-fold enhancement (Figure 2e). Enzyme concentrations remained constant, and adsorption studies confirmed that the enzymes remained in solution, ruling out immobilization effects. In a conventional Oil/Water emulsion system, lipase activity increased up to 4.8-fold using carbon nanotubes and 3.5-fold using carbon nanotubes-gold nanoparticles complex.^36^ These findings indicate that magnetic particles actively modulate enzyme conformation to enhance catalytic function, likely through proximity-induced surface interactions, alterations in hydration shell dynamics, or localized magnetic field effects. To determine whether this effect extends to other enzyme classes, we assessed the activity of L*p*NOX, a water-forming NADH oxidase commonly used in NAD^+^ regeneration. Following incubation with magnetic particles, L*p*NOX displayed a 1.25-fold increase in relative activity (Figure 2e), suggesting that the phenomenon is not limited to lipases and may be applicable to redox enzymes as well. Interestingly, although both LipA and Lip3 showed enhanced activity and structural changes, LipA, which lacks a regulatory lid, underwent more pronounced secondary structure alterations and demonstrated greater activation. This observation supports the hypothesis that the lid in classical lipases provides a degree of structural shielding against environmental perturbations. A previous work already reported here has shown that decreased α-helical content can promote lid opening, while increased β-sheet content enhances structural rigidity and resistance to denaturation.^35^ However, in our study, differential scanning fluorimetry revealed no significant changes in melting temperature for either enzyme after particle exposure (Table S3), indicating that global thermal stability was maintained despite localized structural rearrangements. Together, these results demonstrate that δ-FeOOH magnetic particles can modulate enzyme structure and activity in a concentration-dependent manner, even in the absence of direct immobilization. This preimmobilization effect may offer a tunable strategy to enhance enzyme performance through surface-induced activation and highlights the importance of understanding weak enzyme-support interactions in particle-assisted biocatalytic systems.

### Effect of Enzyme Immobilization on Magnetic Particles on Enzyme Performance

Following the observation that δ-FeOOH magnetic particles can modulate enzyme structure and activity even in the absence of immobilization, we next evaluated how formal immobilization strategies, specifically adsorption and covalent binding, affect enzyme loading and performance, since the support material, and the subsequent possible preparation of the carrier, influence the immobilization parameters.^37^ Enzyme immobilization relies on establishing stable interactions between the enzyme and the support surface, with high surface area materials facilitate greater enzyme retention.^9^ Surface functionalization using cross-linkers such as glutaraldehyde enables covalent attachment by aldehyde groups on the support and surface-exposed amines on the enzyme.^38^ These strong interactions are often used to improve binding stability and reduce enzyme leaching. Compared to typical supports, which reach saturation at enzyme loadings below 50 mg g^–1^ ^19,39^ all magnetic supports tested in this study exhibited significantly higher capacities, consistently exceeding 60 mg g^–1^ (Figure 3a, enzyme loading across supports). This highlights the high binding potential of δ-FeOOH-based materials, particularly when modified for covalent immobilization.

**Figure 3:**
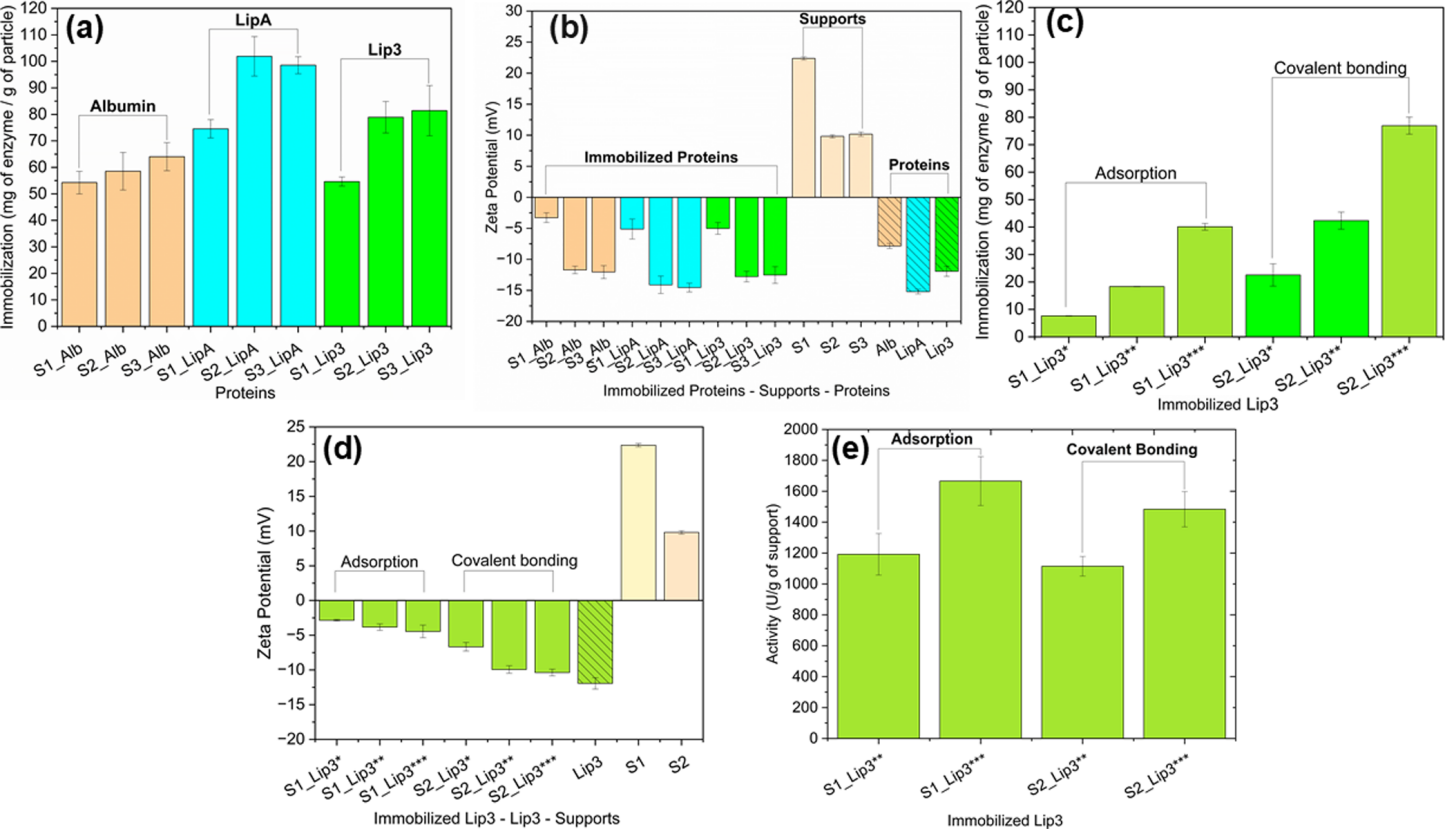
Characterization of albumin and lipase immobilization via adsorption and covalent coupling. Protein loading, surface charge, and catalytic activity were analyzed for immobilized lipase (Lip3) and bovine serum albumin (BSA) across a range of support concentrations and immobilization strategies. Zeta potential measurements were used to monitor progressive surface saturation and protein layer formation. Symbols * to *** indicate increasing amounts of immobilized Lip3 on the support. Protein loading (Figure 3a), zeta potential trends (Figure 3b–d), and enzyme activity (Figure 3e) are shown for both adsorption and covalent binding conditions. Data demonstrate the formation of multilayers at high enzyme concentrations and reveal the relationship between surface charge dynamics and catalytic efficiency. S1—δ-FeOOH@SiO2; S2—δ-FeOOH@SiO2@NH2-GL0.5; S3—δ-FeOOH@SiO2@NH2-GL0.5

Among the functionalized particles, δ-FeOOH@SiO_2_@NH_2_ treated with glutaraldehyde (S2 and S3) supported efficient covalent attachment. However, increasing glutaraldehyde concentration from 0.5% to 1% did not result in further improvement in enzyme loading. This observation is consistent with previous findings by Kaur and Jana,^40^ who reported that while moderate glutaraldehyde concentrations enhance enzyme-support interactions, excessive cross-linking can lead to steric hindrance or limited accessibility, ultimately reducing effective binding. Overcross-linking may also restrict enzyme flexibility or promote nonspecific association with the support surface rather than true covalent immobilization. These results indicate that δ-FeOOH-based magnetic supports are highly effective for both physical adsorption and covalent attachment. However, optimizing cross-linker concentration is critical for maximizing loading efficiency while preserving enzyme accessibility. The high immobilization capacities observed here highlight the potential of these materials for biocatalytic applications that require dense enzyme packing, such as in multienzyme cascade reactions or continuous-flow systems.

### Effect of Increased Enzyme Concentration on Support

Previous studies have shown that high local enzyme concentrations on immobilization supports can lead to reduced catalytic efficiency, often due to steric hindrance or restricted substrate access caused by enzyme crowding.^20^ To monitor how enzyme accumulation progresses during immobilization, we employed zeta potential analysis, a noninvasive technique that sensitively detects changes in surface charge upon enzyme adsorption without requiring chemical modification (Figure 3b–d). As enzyme loading increased, zeta potential measurements revealed a progressive reduction in surface charge, reflecting incremental adsorption of negatively charged lipase molecules. The signal plateaued at approximately 40 mg g^–1^ of immobilized enzyme under covalent binding conditions, reaching a value comparable to that of free lipase in solution (S3_Lip3**, Figure 3d). This suggests that a complete monolayer had formed at this concentration. Notably, further immobilization occurred beyond this point, reaching up to ∼75 mg g^–1^ (S3_Lip3***), indicating multilayer formation (Figure 3c). This additional enzyme retention likely results from protein–protein interactions and tertiary conformational adjustments that promote stacking and secondary adsorption-phenomena previously described in multilayer protein systems.^41–43^ A recent study^19^ in the literature investigating the immobilization of lipase on a diverse surface of magnetic particles via adsorption and covalent binding found results almost ten times lower than the results we report here, achieving a maximum of 16.8 mg/g and results below 2 mg_protein_/g_support_ for some supports. Guilherme et al.^9^ also reported lower results, obtaining values around 7 mg _protein_/g_support_ when studying the immobilization of cellulolytic enzymes on magnetic nanoparticles.

While multilayer formation increases total enzyme loading, it may also reduce catalytic efficiency due to reduced substrate accessibility or occluded active sites. To assess this, we performed activity assays on immobilized lipase samples with similar zeta potential but varying enzyme loads. For adsorption-based immobilization, enzyme loading ranged from 7.46 ± 0.10 to 40.01 ± 1.24 mg g^–1^, while for covalent binding, it ranged from 22.54 ± 4.06 to 76.95 ± 3.10 mg g^–1^. The resulting activities ranged from 1114.61 ± 63.47 to 1665.89 ± 158.27 U g^–1^ (Figure 3e), and immobilized enzymes retained approximately 60% of the specific activity of the free enzyme (Figure 4a). Although increasing enzyme density on the support led to higher total activity, the efficiency per unit enzyme did not scale proportionally. These findings support the occurrence of multilayer formation, where only the enzymes in the first layer maintain optimal catalytic orientation and accessibility. Importantly, despite variations in loading, immobilized lipases maintained their substrate specificity and structural stability (Figure 4a), confirming that the immobilization protocol preserved enzyme function across different concentrations. However, the data also underscores a practical consideration: to avoid diminishing returns and ensure cost-effective biocatalysis, enzyme loading on the support should be optimized to balance total activity with enzyme efficiency. Furthermore, a fundamental point related to enzymes is their stability, as Abellanas-Perez et al.^44^ report that protein–protein interactions can affect enzyme stability, we selected the free and immobilized lipases (Figure 3e) for testing thermal stability and specificity.

**Figure 4:**
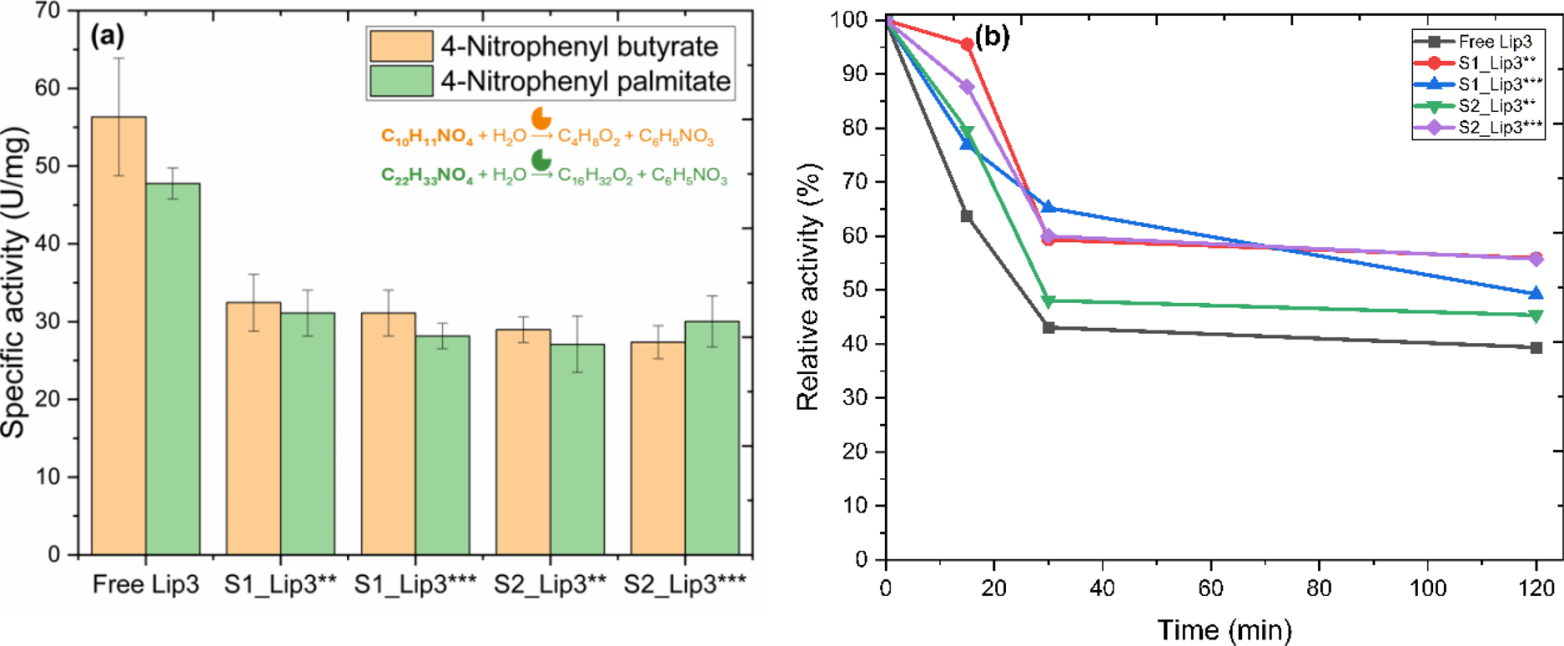
Specificity and thermal stability of free and immobilized Lip3 (a) Substrate specificity and (b) thermal stability of free Lip3 compared to immobilized forms on S1 via adsorption and S2 via covalent bonding at 50 °C for 2 h. S1—δ-FeOOH@SiO2; S2—δ-FeOOH@SiO2@NH2-GL0.5.

### Thermal Stability and Selectivity of Immobilized Lipase

In addition to catalytic activity, enzyme stability is a critical determinant of performance in biocatalytic processes. Previous studies have reported that excessive enzyme loading or crowding can negatively impact stability due to restricted conformational flexibility or unfavorable protein–protein interactions.^41^ However, no such detrimental effects were observed in our system. After 2 h of incubation at 50 °C, free Lip3 retained 39.38% of its initial activity, whereas the immobilized form retained approximately 55% (Figure 4b).

These results indicate that the immobilization strategy not only preserved most of the lipase activity but also conferred enhanced thermal stability. Notably, higher enzyme loading did not impair the stability or specific activity of the immobilized biocatalyst. Instead, increased loading improved the total catalytic output per unit of support, without compromising functional integrity. This finding is particularly relevant for industrial applications, where process efficiency, stability, and support reusability are essential.

## Conclusions

This study presents the first systematic evaluation of δ-FeOOH magnetic particles as a multifunctional support for enzyme immobilization, using lipases, LpNOX, and albumin as model systems. In addition to serving as robust, magnetically recoverable carriers, these particles modulate enzyme conformation and enhance catalytic activity even in the absence of immobilization, revealing a previously underexplored mechanism of surface induced activation. Spectroscopic analyses showed that interaction with δ-FeOOH alters enzyme secondary structure, increasing β-sheet and reducing α-helical content, without compromising thermal stability, suggesting a compact and catalytically favorable conformation, improving enzymes activity. Notably, enzyme activity increased by up to 1.3-fold for both lipases and LpNOX, even without covalent binding. The supports enable multilayer enzyme immobilization at loadings exceeding 60 mg g^–1^ while maintaining enzymatic performance. Zeta potential and circular dichroism confirmed that adsorption continues beyond monolayer saturation (∼40 mg g^–1^) without crowding-induced deactivation. Both adsorption and covalent strategies were effective, with glutaraldehyde functionalized surfaces achieving strong attachment without excessive cross-linking. These findings establish δ-FeOOH as a dual-function platform: a high-capacity immobilization support and a surface-driven modulator of enzyme behavior. As biocatalysis advances toward cascade reactions, continuous processing, and synthetic metabolic networks, materials that can both immobilize and activate enzymes offer new opportunities for optimizing function at the enzyme-material interface.

The main focus of this study was to evaluate how enzyme-particle interactions affect structural and catalytic properties, and therefore, process-related parameters were not explored in detail. Nonetheless, future work should aim to apply this understanding to reactor-based systems for product synthesis, where additional operational factors may influence enzyme performance. Furthermore, assessing enzyme reusability under realistic process conditions would be highly relevant, as it may differ from activity-based reusability measured in controlled assays.

## Supplementary Material



## Notes

Further data regarding protein production, characterization, support characterization and thermal unfolding: Figure S1—Protein concentration, structure, activity, and SDS. Table S1—Lipase information and identification. Table S2—Bacterial strains used for protein production. Table S3—Size, polydispersity index, and zeta potential for supports. Table S4—Thermal unfolding temperature for proteins and physical mixtures (PDF)
