# Supplementary material for: Magnetically Recoverable δ‑FeOOH Particles for Multilayer Enzyme Immobilization and Surface-Induced Activity Tuning
Source: ACS Omega. 2025 Dec 4;10(49):60993–1003. doi: 10.1021/acsomega.5c10160 (PMC12713457; doi:10.1021/acsomega.5c10160)
Supplement: Supplementary file 1 [file ao5c10160_si_001.pdf]

Magnetically Recoverable δ-FeOOH particles for Multilayer Enzyme Immobilization and Surface-Induced Activity Tuning

Francisco Lucas Chaves Almeida[1],[2], Ederson Paulo Xavier Guilherme[2], Maria Isabel Rodriguez-Torres[1], Laura Rotilio[3], Magdalena Malankowska[1], Aliyeh Hasanzadeh[1], Bodil Fliis Holten[4], , Suzana Siebenhaar[3], Lars Michael Skjolding[5], Jens P. Morth[3], John M. Woodley[1], Marcus Bruno Soares Forte*[2], and Elif Erdem*[1]

---

[1] Department of Chemical and Biochemical Engineering, Technical University of Denmark, 2800 Kgs Lyngby, Denmark
[2] Bioprocess and Metabolic Engineering Laboratory, Department of Food Engineering and Technology, School of Food Engineering, University of Campinas, Rua Monteiro Lobato, 80 - CEP 13083-862, Campinas, São Paulo, Brazil
[3] Department of Biotechnology and Biomedicine, Technical University of Denmark, 2800 Kgs Lyngby, Denmark
[4] Department of Chemistry Organic and Inorganic Chemistry, Technical University of Denmark, 2800 Kgs Lyngby, Denmark
[5] Department of Environmental and Resource Engineering, Technical University of Denmark, 2800 Kgs Lyngby, Denmark
Corresponding author email: forte@unicamp.br / eliferd@kt.dtu.dk

SUPPLEMENTARY MATERIAL

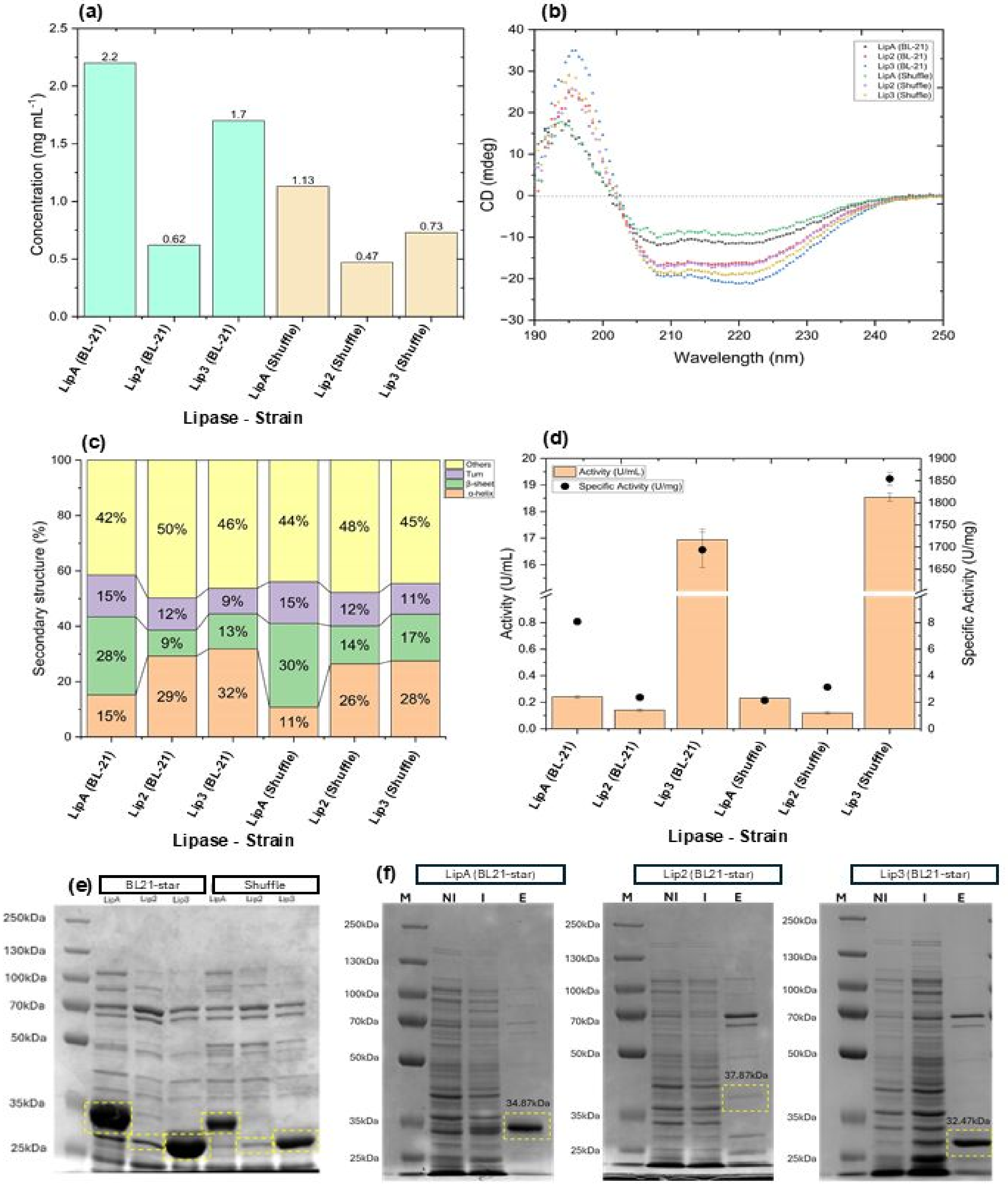

Figure S1 – Protein concentration (a)**,** circular dichroism (CD) spectra (b), secondary structure (c), activity (d) and SDS of lipases (e-f)

M – Ladder, **NI** – Non-Induced, **I** – Induced, **E** – Eluted enzyme

Table S1 – Lipases information and identification

Lipase	GenBank code	GenPept Identical Proteins Graphics
LipA<br>ε 21430 M^{-1} Cm^{-1}<br>MW 34,9 KDa	MDZ5722820.1	>MDZ5722820.1 lipoyl synthase, partial [Bacillus sp. SXabc123]<br>LAKKDEHLRKPEWLKIKLNTNENYTGLKKLMRENNLHTVCEEAKCPNIHEC<br>WAVRRTATFMILGSVCTRACRFCAVKTGLPTELDLQEPERVADSVALMNLK<br>HAVITAVARDDQKDGGAGIFAETVRAIRRKSPFTTIEVLPSDMGGNYDNLKT<br>LMDTRPDILNHNIETVRRLTPRVRARATYDRSLEFLRRAKEMQPDIPTKSSIM<br>IGLGETKEEIIEVMDDLLANNVDIMAIGQYLQPTKKHLKVQKYYHPDEFAEL<br>KEIAMQKGFSHCEAGPLVRSS
Lip2<br>ε 27850 M^{-1} Cm^{-1}<br>MW 32,5 KDa	CAB91111.1	>CAB91111.1 triacylglycerol lipase [Yarrowia lipolytica]<br>MKLSTILFTACATLAAALPSPITPSEAAVLQKRVYTSTETSHIDQESYNFFEK<br>YARLANIGYCVGPGTKIFKPFNCGLQCAHFPNVELIEEFHDPRLIFDVSGYLA<br>VDHASKQIYLVIRGTHSLEDVITDIRIMQAPLTNFDLAANISSTATCDDCLVH<br>NGFIQSYNNTYNQIGPKLDSVIEQYPDYQIAVTGHSLGGAAALLFGINLKVN<br>GHDPLVVTLGQPIVGNAGFANWVDKLFFGQENPDVSKVSKDRKLYRITHRG<br>DIVPQVPFWDGYQHCSGEVFIDWPLIHPPLSNVVMCQGQSNKQCSAGNTLL<br>QQVNVIGNHLQYFVTEGVCGI
Lip3<br>ε 35870 M^{-1} Cm^{-1}<br>MW 37,6 KDa	ACC76759.1	>ACC76759.1 lipase [Proteus sp. K107]<br>MSTKYPIVLVHGLAGFNEIVGFPYFYGIADALRQDGHQVFTASLSAFNSNEV<br>RGKQLWQFVQTLLQETQAKVNFIGHSQGPLACRYVAANYPDSVASVTSING<br>VNHGSEIADLYRRIMRKDSIPEYIVEKVLNAFGTIISTFSGHRGDPQDAIAALE<br>SLTTEQVTEFNNKYPQALPKTPGGEGDEIVNGVHYYCFGSYIQGLIAGEKGN<br>LLDPTHAAMRVLNTFFTEKQNDGLVGRSSMRLGKLIKDDYAQDHIDMVNQ<br>VAGLVGYNEDIVAIYTQHAKYLASKQL
LpNOX<br>ε 44810 M^{-1} Cm^{-1}<br>MW 49,4 Kda	-	N-His6x-LpNOX (reverse on plasmid map) [Lactobacillus pentosus]<br>MKVIVIGCTHAGTAAVNQILASNPETDVTIYERNDNVSFLSCGIALYLGGEV<br>ADPQGLFYSSPEQLAKLGANVHMQHDVTDVDTENHEITVTDLKTGESKKD<br>YYDKLVVTTGSWPVIPPIDGIDSPNVYLCKNWTHAQSLWEAAKPAKRVIVI<br>GGGYIGTELVEAYQKQGKEVTLIDGLPRILNKYLDKGFTDRVEKDFVDHGI<br>KMALNQMVKGFSDDGKEVTVKTDKGSYTADMAILCVGFRPNTSLLKGKV<br>DMNPNGSIKTNDYMQTSDPDIYGAGDSVAVHYNPTKKDAYIPLATNAVRQ<br>GTLVGLNIFKPTRKYMGTQSTSGLMLFGKTIVSSGMTLEHAQAEKVPAEAV<br>TFEDNYRPEFMPTTKPVLMQLVYNPETREILGAQFMSEHDVSQSANVISVMI<br>QNHNTIDDLGFVDMFFQPIYDRPFNYLNLLGQAAIAHAAEKVTE

Table S2 – Bacterial strains used for protein production

**Strain/Plasmid**	**Description**	**Reference**
Escherichia coli<br>BL-21star_LipA	F^-*ompT* *hsd*SB (r_B–, m_B–) *gal dcm rne*131 (DE3)<br>Pet22b 6xHis tag from N-termini	This work
Escherichia coli<br>BL-21star_Lip3	F^-*ompT* *hsd*SB (r_B–, m_B–) *gal dcm rne*131 (DE3)<br>Pet22b 6xHis tag from N-termini	This work
Escherichia coli<br>Shuffle_LipA	MiniF *lysY* (Cam^R) */ fhuA2 lacZ::T7 gene1 [lon] ompT ahpC gal λatt::pNEB3-r1-cDsbC* ($Spec^R$, *$lacI^q$*) *ΔtrxB sulA11 R(mcr-73::miniTn10*--Tet^S*)2 [dcm] R(zgb-210::Tn10* --Tet^S*) endA1 Δgor Δ(mcrC-mrr)114::IS10*<br>Pet22b 6xHis tag from N-termini	This work

Escherichia coli Shuffle_Lip3	MiniF *lysY* (Cam^R) / *fhuA2 lacZ::T7 gene1 [lon] ompT ahpC gal λatt::pNEB3-r1-cDsbC* (Spec^R, *lacI^q*) *ΔtrxB sulA11 R(mcr-73::miniTn10*--Tet^S*)2 [dcm] R(zgb-210::Tn10* --Tet^S*) endA1 Δgor Δ(mcrC-mrr)114::IS10* Pet22b 6xHis tag from N-termini	This work

Table S3 – Size, Polydispersity index and zeta potential for supports

Support	Z-average –(d.nm)	PDI	Zeta Potential (mV)
δ-FeOOH@SiO_2@NH_2	2677 ± 333.6	0.20 ± 0.06	22.4 ± 0.31
δ -FeOOH@SiO_2@NH_2-GL0.5	2573 ± 113.7	0.25 ± 0.01	9.81 ± 0.31
δ -FeOOH@SiO_2@NH_2-GL1	2342 ± 106	0.27 ± 0.05	10.2 ± 0.41

Table S4 – Thermal unfolding temperature (Tm) for proteins and physical mixtures

Protein	Tm (°C)
Alb	60.49
Alb (370ug)	60.67
Alb (1860ug)	60.31
LipA	50.42
LipA (370ug)	48.46
LipA (1860ug)	48.43
Lip3	48.64
Lip3 (370ug)	48.83
Lip3 (1860ug)	48.65
